# ADAM17 is an HIV-1 Restriction Factor Antagonized by Nef

**DOI:** 10.21203/rs.3.rs-6923186/v1

**Published:** 2025-06-25

**Authors:** Yong-Hui Zheng, Qiuchen Li, Sunan Li, Bowei Ye, Jie Liang

**Affiliations:** University of Illinois Chicago; University of Illinois Chicago; Harbin Veterinary Research Institute; University of Illinois Chicago; University of Illinois at Chicago

**Keywords:** Restriction factor, ADAM17, TACE, Nef, HIV-1 Env

## Abstract

Nef is an HIV-1 accessory protein critical to viral pathogenesis. While its role in immune evasion is well established, the mechanism by which Nef enhances virion infectivity remains incompletely understood. Here, we identify ADAM17 (TACE) as a host restriction factor through affinity purification–mass spectrometry. ADAM17 associates with HIV-1 Env in the endoplasmic reticulum (ER), reducing Env expression and incorporation into virions, leading to an approximately 100-fold reduction in infectivity. This restriction is independent of ADAM17’s metalloprotease activity and instead depends on its prodomain, which alters Env intracellular trafficking and broadly inhibits both laboratory-adapted and circulating HIV-1 strains. Nef counteracts this restriction by decreasing cellular ADAM17 levels through Rab11^+^ endosome– and CD63^+^ exosome–mediated extracellular export. Given ADAM17’s known role in TNF signaling, these findings reveal a previously unrecognized link between Nef-induced pro-inflammatory responses and evasion of host restriction during HIV-1 pathogenesis.

## Introduction

HIV-1 encodes a Negative Factor (Nef) protein that enhances virion infectivity and promotes immune evasion, accelerating progression to AIDS. Nef primarily localizes to the inner leaflet of the plasma membrane and boosts viral replication, though the mechanisms underlying this enhancement remain incompletely understood. One well-characterized function of Nef is to downregulate serine incorporator 5 (SERINC5), which incorporates into virions from the cell surface and inhibits viral entry^1,2^. SERINC5 contains 10 transmembrane domains organized into two subdomains and shares a conserved tertiary structure with non-ATP-dependent lipid transporters^3^. It is polyubiquitinated in the trans-Golgi network (TGN) by the Cullin3-KLHL20 E3 ligase through K33/K48-branched ubiquitin chains and trafficked to the plasma membrane^4^. Nef counteracts SERINC5 by inducing its phosphorylation via the Cyclin K/CDK13 complex and promoting its internalization through receptor-mediated endocytosis, leading to lysosomal degradation^5,6^. Nonetheless, SERINC5 selectively inhibits laboratory-adapted HIV-1 strains, but not those primary isolates^7–9^. In addition, while SERINC5 exhibits scramblase activity that exposes phosphatidylserine on the virion surface^10^, this activity is dispensable for its antiviral function^11^, leaving its exact antiviral mechanism unclear. Notably, Nef-deficient (ΔNef) HIV-1 remains strongly restricted in certain human *SERINC5*-knockout CD4^+^ T cells^12,13^, suggesting the existence of an additional anti-HIV-1 factor targeted by Nef.

A disintegrin and metalloprotease (ADAM) family of proteins regulates the shedding of surface receptors, cytokines, and growth factors^14^. ADAM17, also known as TNF-α converting enzyme (TACE), is responsible for cleaving pro-TNF-α to release its active, pro-inflammatory form. Here, we identify ADAM17 as a potent inhibitor of HIV-1 infectivity through its targeting of the envelope (Env) glycoprotein—a restriction counteracted by Nef.

## Results

### ADAM17 is a potent anti-HIV-1 host factor antagonized by Nef

**Figure 1:**
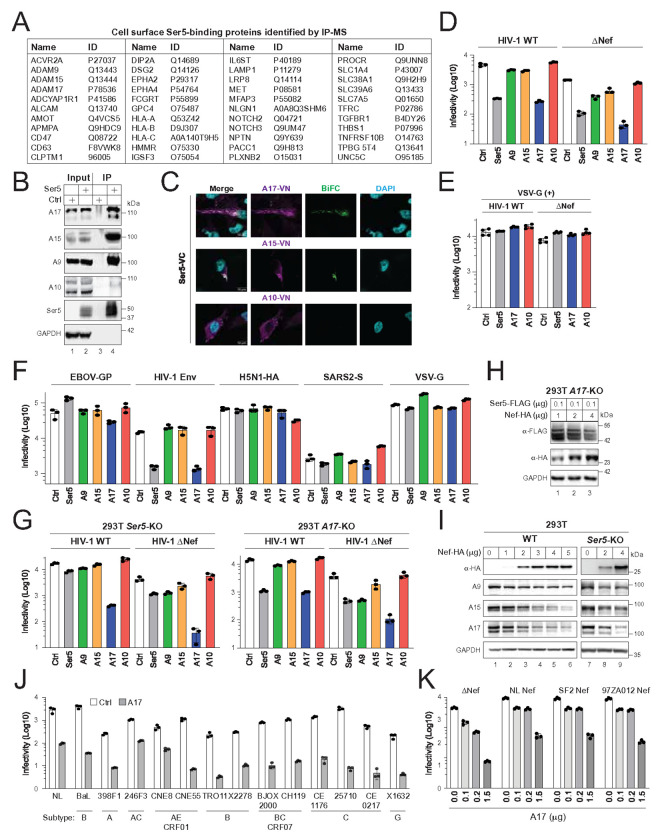
Identification of ADAM17 as Nef-sensitive anti-HIV-1 host factor. (**A**) FLAG-tagged Ser5 was expressed in HEK293T cells and immunoprecipitated using anti-FLAG beads. Bead-associated proteins were identified by mass spectrometry, and the cell surface proteins are shown. (**B**) Ser5-FLAG was expressed in HEK293T cells and immunoprecipitated (IP) with anti-FLAG beads. ADAM (A9, A10, 15, A17) proteins were detected with their specific antibodies by Western blotting (WB). (**C**) Indicated ADAM proteins with a C-terminal VN-HA-tag were expressed with Ser5 with a C-terminal FLAG-VC-tag in HeLa cells. ADAM proteins were stained with anti-HA followed by fluorescent 2^nd^ antibodies, and nuclei were stained with DAPI. BiFC signals were determined by confocal microscopy (scale bar 10 μm). (**D**) HIV-1 (NL) wild-type (WT) and Nef-deficient (ΔNef) viruses were produced from HEK293T cells in the presence of the indicated host protein expression vector at a ratio as in Fig. S3A. Virion infectivity was measured after infection of TZM-bI cells. (**E**) HIV-1 (NL) viruses were produced in the presence of indicated host proteins and pseudotyped with VSV-G, and virion infectivity was analyzed after infection of TZM-bI cells. (**F**) ΔNef HIV-1 Luc-reporter pseudovirions (PVs) expressing indicated viral glycoproteins were produced from HEK293T cells in the presence of indicated ectopic host proteins. Virion infectivity was determined after infection of different target cells. **(G)** HIV-1 (NL) viruses were produced from HEK293T *Ser5*-KO and *A17*-KO cells in the presence of the indicated host factors. Virion infectivity was analyzed in TZM-bI cells. **(H)** Ser5 was expressed with increasing amounts of Nef (NL) in HEK293T *A17*-KO cells, and their expression was determined by WB. **(I)** HEK293T WT and *Ser5*-KO cells were transfected with increasing amounts of Nef (NL) expression vector. The endogenous ADAM protein expression was determined by WB. **(J)** Replication-competent HIV-1 proviruses expressing indicated Envs from global isolates were expressed with A17 in HEK293T cells. Virion infectivity was analyzed in TZM-bI cells. **(K)** HIV-1 (NL) proviruses expressing indicated Nef proteins were expressed with increasing amounts of A17 in HEK293T cells. Virion infectivity was analyzed in TZM-bI cells. All experiments were repeated three times, and representative results are shown.

To investigate the antiviral mechanism of SERINC5 (Ser5), we analyzed Ser5-interacting proteins at the cell surface using mass spectrometry. Among 44 identified candidates were three ADAM family members—ADAM9 (A9), ADAM15 (A15), and ADAM17 (A17) (Fig. 1A). These hits were confirmed in another independent experiment (**Fig. S1**). Co-immunoprecipitation experiments validated that Ser5 pulls down endogenous A9, A15, and A17, but not ADAM10 (A10), which was absent from the proteomics data (Fig. 1B). Likewise, ectopically expressed A17, but not A10, co-immunoprecipitated with Ser5; and A17 pulled down Ser5, whereas GFP did not (**Fig. S2**).

We further validated these interactions in live cells using bimolecular fluorescence complementation (BiFC). When ADAM proteins with a C-terminal VN-HA tag were expressed with Ser5 fused to a FLAG-VC tag in HeLa cells, BiFC signals confirmed interactions between A17–Ser5 and A15–Ser5, but not A10–Ser5 (Fig. 1C). We could not detect BiFC signal from A9–Ser5 due to a proteolytic cleavage that removed the A9 VN-HA tag (see below).

To assess antiviral activity, we expressed Ser5 or ADAM proteins with the HIV-1 NL strain (wild-type or ΔNef) in HEK293T cells. Expression plasmid amounts were adjusted to normalize protein levels (**Fig. S3A**). While expression of A9 showed cleavage (loss of a ~25 kDa C-terminal fragment), viral particle production was largely unaffected by any construct (**Fig. S3B**). Infectivity assays using TZM-bl cells revealed that Ser5 suppressed wild-type (WT) and ΔNef virion infectivity ~10-fold, as expected (Fig. 1D). While A10 had no effect, A9 and A15 reduced WT virion infectivity <2-fold and ΔNef virion infectivity <3-fold (Fig. 1D). In contrast, A17 reduced WT virion infectivity ~15-fold and ΔNef virion infectivity ~35-fold (Fig. 1D), indicating that A17 has a strong antiviral activity that can be counteracted by Nef.

To test whether A17 blocks HIV-1 entry, we used pseudotyped HIV-1 with vesiculovirus (VSV)-glycoprotein (G)—a glycoprotein known to overcome Ser5 restriction. VSV-G pseudotyping largely abolished A17-mediated restriction (Fig. 1E). In addition, using HIV-1 ΔNef luciferase pseudoviruses (PVs) bearing various glycoproteins—HIV-1 Env, Ebola virus (EBOV) glycoprotein (GP), influenza A virus (H5N1) hemagglutinin (HA), SARS-CoV-2 (SARS2) spike (S), or VSV-G—we found that A17 and Ser5 inhibited PVs only expressing HIV-1 Env (Fig. 1F). A9, A10, and A15 had no effect. Thus, A17 specifically inhibits HIV-1 infection at viral entry.

To assess whether A17 and Ser5 are functionally associated, we generated HEK293T knockout (KO) cell lines using CRISPR. *Ser5*-KO was confirmed by next-generation sequencing (NGS) (**Fig. S4**); *A17*-KO was validated by Western blotting (**Fig. S5A**). In *Ser5*-KO cells, A17 retained strong antiviral activity and reduced ΔNef virion infectivity up to 100-fold, whereas the Ser5 inhibition remained consistent (Fig. 1G, left). In *A17*-KO cells, both Ser5 and A17 suppressed virion infectivity similarly to wild-type cells (Fig. 1G, right). Furthermore, Nef still downregulated Ser5 in *A17*-KO cells (Fig. 1H), and it also reduced the endogenous A9, A15, and A17 expression in *Ser5*-KO cells (Fig. 1I). These results indicate that A17 and Ser5 restrict HIV-1 through independent pathways.

To determine the breadth of A17’s antiviral activity, we tested its effect on HIV-1 subtypes A, B, C, and G, and circulating recombinant forms (CRFs) AE and BC. A17 restricted all tested strains to a similar degree as the NL reference strain (Fig. 1J). Nef proteins from other strains—SF2 (subtype B) and 97ZA012 (subtype C)—also effectively counteracted A17 (Fig. 1K). Thus, A17-mediated HIV-1 restriction and Nef antagonism are subtype-independent.

### ADAM17 inhibits HIV-1 Env expression

**Figure 2:**
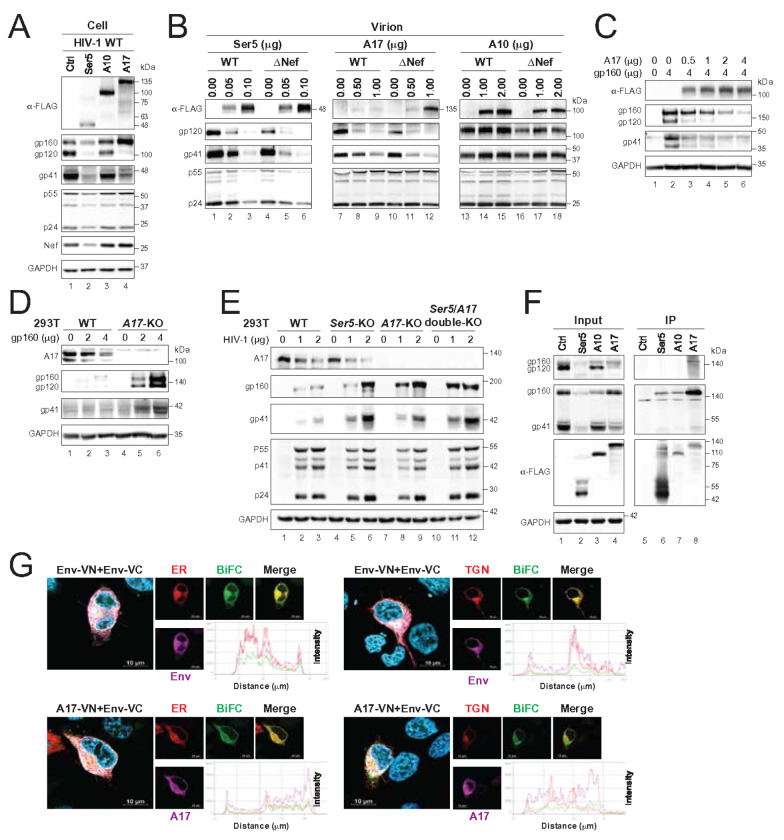
ADAM17 inhibits HIV-1 Env expression. **(A)** HEK293T cells were transfected with HIV-1 (NL) proviral vector and the indicated host protein expression vector. Viral protein expression was determined by WB. (**B**) HIV-1 (NL) WT and ΔNef viruses were produced from HEK293T cells in the presence of increasing amounts of indicated host factors. Virions were purified by ultracentrifugation and analyzed by WB. **(C)** HIV-1 gp160 was expressed with increasing amounts of A17 in HEK293T *A17*-KO cells. Protein expression was determined by WB. **(D)** HEK293T WT and *A17*-KO cells were transfected with increasing amounts of an HIV-1 gp160 expression vector. Protein expression was determined by WB. **(E)** HEK293T WT and indicated KO cells were transfected with increasing amounts of HIV-1 (NL) proviral vector. Protein expression was determined by WB. **(F)** HIV-1 gp160 was expressed with the indicated host proteins with a FLAG-tag in HEK293T cells. Proteins were immunoprecipitated using anti-FLAG beads and analyzed by WB. **(G)** Env-VN-HA or A17-VN-HA was expressed with Env-FLAG-VC and the mCherry-tagged ER marker calreticulin (CALR) or Golgi marker TGN46 in HeLa cells. Their localization was tracked by BiFC using confocal microscopy. Env-VN-HA and A17-VN-HA were stained with an anti-HA antibody to confirm the specificity of the BiFC signals (scale bar 10 μm). All experiments were repeated three times, and representative results are shown.

To investigate the A17 antiviral mechanism, we examined its effect on HIV-1 structural protein expression. In HEK293T cells expressing HIV-1 provirus with Ser5, A10, or A17, we found that A10 did not affect Gag (p55, p24), Env (gp120, gp41), or Nef levels (Fig. 2A, lane 3). Ser5 reduced the expression of all three viral proteins (lane 2), while A17 selectively suppressed gp120 and gp41 without affecting Gag or Nef (lane 4).

We then assessed viral protein levels in WT and ΔNef virions. Ser5 and A17 were more abundant in ΔNef virions than in WT (Fig. 2B, lanes 2–3, 5–6, 8–9, 11–12). As expected, A10 did not affect Env incorporation, and its levels were not affected by Nef (lanes 13–18). While Ser5 reduced Env levels, this was associated with a corresponding drop in Gag. In contrast, A17 specifically and robustly reduced gp120 and gp41, especially in ΔNef virions (lanes 7–12), indicating that A17 suppresses Env incorporation by limiting its cellular expression—an effect counteracted by Nef.

To investigate this further, we expressed HIV-1 gp160 in *A17*-KO cells alongside increasing amounts of A17. We observed a dose-dependent reduction in gp160, gp120, and gp41 levels (Fig. 2C). Notably, a significantly higher amount of A17 was required to reduce gp160 compared to gp120 and gp41, which may explain why the reduction of gp160 was not apparent in the previous experiment (Fig. 2A, lane 4). Endogenous A17 activity was confirmed by comparing Env levels in WT and *A17*-KO cells: gp160, gp120, and gp41 were significantly higher in KO cells (Fig. 2D). Interestingly, Env also suppressed endogenous A17 expression in a dose-dependent manner (lanes 1–3).

We also analyzed Env expression in *Ser5*-KO and *Ser5*/ *A17* double-KO cells. Env levels increased in *Ser5*-KO cells but were elevated even more in *A17*-KO and double-KO cells, especially at lower provirus input (Fig. 2E). Gag expression remained relatively unaffected, highlighting A17’s selective targeting of Env. These results demonstrate that both Ser5 and A17 inhibit Env expression by targeting its gp160 precursor, with A17 exerting a more potent effect.

Next, we tested whether A17 physically interacts with Env. FLAG-tagged Ser5, A10, or A17 were expressed with Env in HEK293T cells, and immunoprecipitation (IP) was performed using anti-FLAG beads. A17 pulled down gp160, detected with both anti-gp41 and anti-gp120 antibodies (Fig. 2F, lane 8). Ser5 and A10 also pulled down gp160, but signals were weaker and only detectable with the more sensitive anti-gp41 antibody (lanes 6–7), suggesting a stronger and/or more stable interaction between A17 and gp160.

We further examined A17–Env interactions in live cells using BiFC. In a previously validated BiFC assay for Env oligomerization^9^, expression of Env-VN and Env-VC with mCherry-tagged ER (calreticulin) or Golgi (TGN46) markers confirmed that Env localizes to both compartments (Fig. 2G, top panels). When A17-VN and Env-VC were expressed together, BiFC signals confirmed complex formation (bottom panels). However, the A17–Env BiFC signal colocalized strongly with the ER marker but not the Golgi marker, suggesting that A17 traps Env in the ER and disrupts its trafficking to the Golgi, thereby inhibiting its expression and maturation.

### ADAM17 inhibits HIV-1 Env expression via prodomain

**Figure 3:**
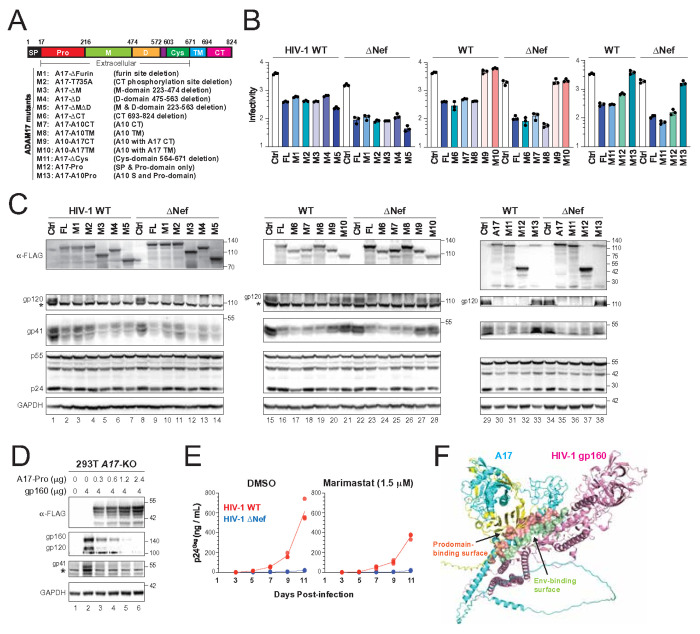
ADAM17 inhibits HIV-1 Env expression via prodomain. **(A)** A schematic illustration of the A17 protein is shown on the top. A total of 13 ADAM17 mutants (M1-M13) were created to map the anti-HIV-1 region. Pro, prodomain; M, metalloproteinase domain; D, disintegrin domain; Cys, cysteine-rich domain; TM, transmembrane domain; CT, cytoplasmic tail. **(B)** HIV-1 (NL) viruses were produced from HEK293T cells in the presence of A17 mutants, and virion infectivity was analyzed in TZM-bI cells. **(C)** HIV-1 (NL) WT and ΔNef proviruses were expressed with A17 mutants in HEK293T cells. Protein expression was determined by WB. **(D)** HIV-1 gp160 was expressed with increasing amounts of A17-Pro in HEK293T *A17*-KO cells. Protein expression was determined by WB. An unspecific band is indicated by a star (*). **(E)** MOLT-3 cells were infected with HIV-1 (NL) and treated with a metalloproteinase inhibitor Marimastat. Viral growth curves were measured by p24^Gag^ ELISA. **(F)** Predicted A17-gp160 complex structure is shown. A17 prodomain is shown in yellow, with its interacting surface highlighted in red. The remainder of the A17 structure is displayed in cyan. HIV-1 gp160 is shown in pink, and the region interacting with the prodomain is rendered as a green surface. All experiments were repeated three times, and representative results are shown.

Human A17 is an 824-amino-acid protein composed of a signal peptide (SP), prodomain (Pro), metalloprotease (M) domain, disintegrin (D) domain, cysteine-rich (Cys) domain, transmembrane (TM) domain, and a C-terminal cytoplasmic tail (CT) (Fig. 3A). A17 is synthesized as an inactive proenzyme in the ER and activated in the TGN through two sequential furin cleavages that remove the prodomain. The mature, active form then traffics to the plasma membrane and cleaves membrane-bound substrates.

To dissect which domains are essential for A17’s antiviral activity, we generated 13 deletion and chimeric mutants (Fig. 3A), including M1, M3–M5: A17 constructs with deletions in the furin-cleavage site, M, and/or D domains; M2, M6: A17 with either a CT deletion or a T735A point mutation that disrupts dimerization; M7, M8: A17 containing the CT or TM domain of A10; M9, M10: A10 containing the CT or TM domain of A17; M11: A17 with a Cys domain deletion; M12: A17 prodomain only; and M13: A17 containing the SP and prodomain of A10. Constructs M1–M8, M11, and M12 retained full antiviral activity, while M9, M10, and M13 had no detectable effect—similar to A10 or vector control (Ctrl) (Fig. 3B).

We next tested how these mutants affected Env expression. All mutants were expressed at comparable levels (Fig. 3C). Consistent with their antiviral activity, M9, M10, and M13 failed to suppress gp41 and gp120 expression in both WT and ΔNef backgrounds (Fig. 3C, lanes 20–21, 27–28, 33, 38). Gag levels remained largely unaffected across all mutants.

These results indicate that the A17 prodomain is necessary and sufficient for antiviral activity. The prodomain-only mutant (M12) retained activity, while the A10 prodomain in M13 did not. In addition, dimerization is not required, as neither CT deletion (M6) nor the T735A mutation (M2) impaired function. Moreover, proteolytic cleavage of the prodomain is not required, as deletion of the furin-cleavage site (M1) had no effect.

To confirm that the prodomain alone is sufficient to inhibit Env, we expressed M12 in *A17*-KO HEK293T cells along with HIV-1 Env. The prodomain dose-dependently suppressed gp160, gp120, and gp41 expression (Fig. 3D).

Because mutants M3 and M5 retained antiviral activity, we further tested whether marimastat, a broad-spectrum metalloprotease inhibitor, affects HIV-1 replication. Human MOLT-3 T cells were infected with WT or ΔNef virus and treated with 1.5 μM marimastat. The WT virus replication was slightly suppressed, and the poor ΔNef replication was not affected (Fig. 3E). Thus, we concluded that A17’s metalloprotease activity is dispensable for its anti-HIV-1 activity.

To investigate how the A17 prodomain interacts with HIV-1 gp160 and inhibits its expression, we predicted the structure of the A17–gp160 complex using AlphaFold3. The model confirms that A17 binds gp160 through its prodomain (Fig. 3F). In addition, it reveals that the prodomain specifically binds the gp41 ectodomain, including five residues within the membrane-proximal external region (MPER) (Fig. 3F, **Table S1**). Collectively, these findings demonstrate that A17 inhibits HIV-1 Env expression through a non-proteolytic mechanism that depends solely on its prodomain.

### Eliminating the requirement for Nef by knocking out *ADAM17* and *SERINC5*

**Figure 4:**
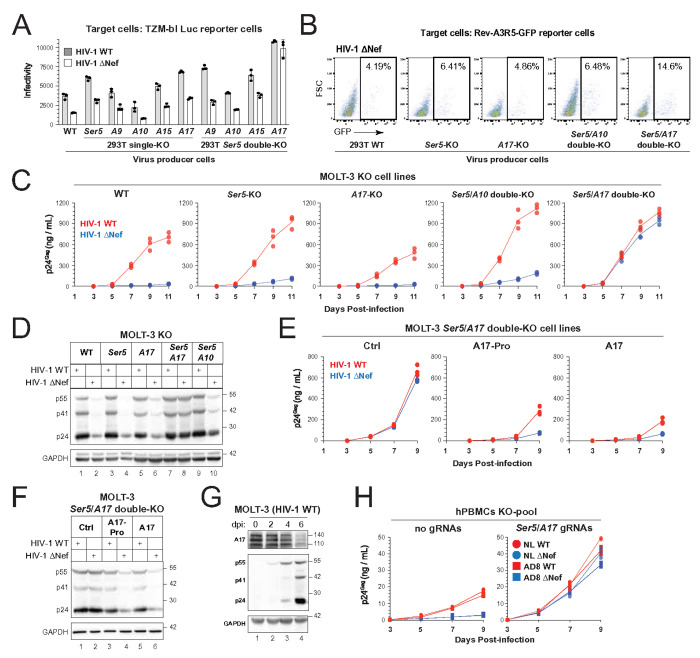
Elimination of the requirement of Nef for HIV-1 infection by knocking out *ADAM17* and *SERINC5*. **(A)** HIV-1 (NL) viruses were produced from HEK293T WT and indicated single- or double-KO cells. Virion infectivity was analyzed in TZM-bI cells. **(B)** HIV-1 (NL) ΔNef viruses were produced from HEK293T WT and indicated single- or double-KO cells. Virion infectivity was analyzed in Rev-A3R5-GFP reporter CEM CD4^+^ T cells. **(C)** MOLT-3 and its indicated KO cells were infected with HIV-1 (NL). Viral growth curves were measured by p24^Gag^ ELISA. **(D)** Eight days post-infection, viral protein expression in (C) was determined by WB. **(E)** MOLT-3 *Ser5*/ *A17* double-KO cells were reconstituted with A17 or A17-Pro and infected with HIV-1 (NL). Viral growth curves were measured by p24^Gag^ ELISA. **(F)** Nine days post-infection, viral protein expression in (E) was determined by WB. **(G)** MOLT-3 cells were infected with HIV-1 (NL) WT viruses. The A17 and HIV-1 Gag expression were determined by WB. Dpi: days post-infection. **(H)** PHA-activated hPBMCs were nucleofected with Cas9 RNPs containing *A17* and *Ser5* guide (g) RNAs. Cells were infected with CXCR4-tropic (NL) or CCR5-tropic (AD8) WT or ΔNef HIV-1. Viral growth curves were determined by p24^Gag^ ELISA. All experiments were repeated three times, and representative results are shown.

To assess the contribution of endogenous ADAM proteins to HIV-1 restriction, we individually knocked out *A9*, *A10*, and *A15* in HEK293T cells, confirming each knockout by WB (**Fig. S5A**). HIV-1 infectivity assays in TZM-bl cells revealed no change in viral infectivity from *A10*-KO cells, confirming A10 lacks antiviral activity (Fig. 4A). In contrast, *A9*-KO and *A15*-KO modestly increased infectivity, indicating weaker antiviral roles. Infectivity was more strongly enhanced in viruses produced from *Ser5*-KO and *A17*-KO cells, highlighting their more potent restriction. However, even in single knockouts, WT virus remained significantly more infectious than ΔNef virus, suggesting Nef continued to counteract another host restriction factor.

To explore possible redundancy, we generated *Ser5* double-KO cell lines with *A9*, *A10*, *A15*, or *A17* (**Fig. S5A**). In *Ser5*/*A9*, *Ser5*/*A10*, and *Ser5*/*A15* double-KOs, Nef still enhanced infectivity (Fig. 4A). However, in *Ser5*/*A17* double-KO cells, both WT and ΔNef viruses showed equivalent, elevated infectivity, indicating that Nef is no longer required when Ser5 and A17 are not expressed.

We further confirmed this by infection of human CEM-derived Rev-GFP reporter T cells. ΔNef virus produced from *Ser5*/*A17* double-KO HEK293T cells showed nearly 3-fold higher infectivity compared to ΔNef virus from single-KO or *Ser5*/*A10* double-KO cells (Fig. 4B).

To extend these findings to T cells, we generated *Ser5*, *A17*, and double KO MOLT-3 cells. A10 was also knocked out for the control comparison. KO validations were performed by NGS (Ser5), Western blot (A10, A17), and flow cytometry (A10, A17) (**Figs. S4–S5**). Viral growth curves over 11 days showed robust replication of WT virus across all lines (Fig. 4C). In contrast, ΔNef virus replicated poorly in WT and *A17*-KO cells and only slightly better in *Ser5*-KO and *Ser5*/*A10* double-KO cells. However, in *Ser5*/*A17* double-KO cells, ΔNef virus replicated robustly—reaching WT levels. Western blot confirmed equal Gag expression between WT and ΔNef infections in these cells (Fig. 4D, lanes 7–8).

To validate the antiviral role of A17, we reconstituted A17 or its prodomain alone in *Ser5*/*A17* double-KO MOLT-3 cells (**Fig. S6**). Both suppressed WT and ΔNef virus infection, restoring the requirement for Nef to enhance infectivity (Fig. 4E, Fig. 4F). Moreover, A17 expression was downregulated upon productive WT infection (Fig. 4G), consistent with Nef-mediated antagonism.

Finally, we evaluated this mechanism in primary cells. Human peripheral blood mononuclear cells (hPBMCs) were activated with phytohemagglutinin (PHA) and necleofected with Cas9-GFP ribonucleoproteins (RNPs) targeting *A17* and *Ser5*. Roughly 33% of cells were GFP^+^ (**Fig. S7A**), and after sorting out these GFP^+^ cells, ~50% of them lost surface A17 (**Fig. S7B**). These GFP^+^ cells were then infected with HIV-1 CXCR4-tropic (NL) and CCR5-tropic (AD8) strains. In the absence of guide RNAs, both NL and AD8 WT viruses outperformed their ΔNef viruses, as expected (Fig. 4H). However, in *A17*/*Ser5*-edited cells, these WT and ΔNef viruses replicated equally and efficiently, mirroring the double-KO HEK293T and MOLT-3 phenotype.

Collectively, these findings demonstrate that Nef’s essential function during HIV-1 infection is to counteract the antiviral activities of SERINC5 and ADAM17.

### Nef antagonizes ADAM17 by extracellular secretion

**Figure 5:**
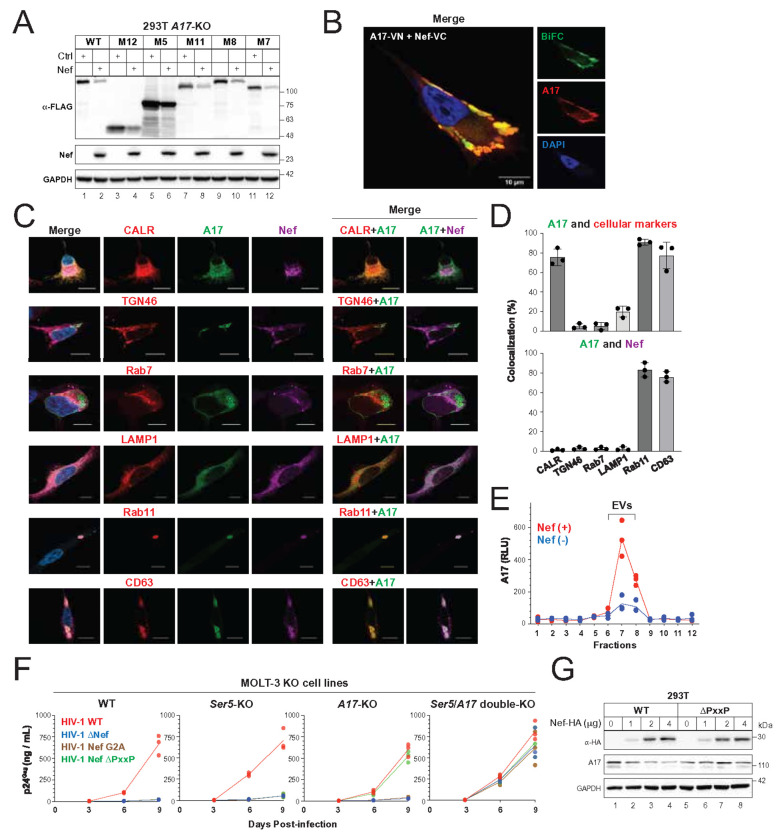
Nef decreases ADAM17 cellular expression via extracellular secretion. **(A)** Nef was expressed with indicated A17 mutants in HEK293T *A17*-KO cells, and their expression was determined by WB. **(B)** A17-VN-HA and Nef-FLAG-VC were expressed in HeLa cells, and the A17-Nef complex was tracked by BiFC. A17-VN-HA was stained with an anti-HA antibody to validate the BiFC signals (scale bar 10 μm). **(C)** Indicated mCherry-tagged cellular markers were expressed with GFP-tagged A17 and HA-tagged Nef in HeLa cells. Nef was stained with an anti-HA antibody. Their colocalization was determined by confocal microscopy (scale bar 10 μm). **(D)** The colocalization of A17 with cellular markers or Nef was statistically analyzed, and the quantification is shown. **(E)** A17-HiBiT was expressed with Nef in HEK293T cells. Extracellular vesicles (EVs) were purified from the supernatants by SEC. A17 levels in these fractions were determined by measuring the NLuc activity. **(F)** Indicated MOLT-3 KO cell lines were infected with HIV-1 (NL) WT and indicated Nef mutant viruses. Viral growth was determined by p24^Gag^ ELISA. **(G)** HEK293T cells were transfected with increasing amounts of Nef (NL) WT and ΔPxxP mutant expression vectors. The endogenous A17 expression was determined by WB. • All experiments were repeated three times, and representative results are shown.

All active A17 mutants retaining the prodomain remained sensitive to Nef (Fig. 3B). To confirm that the Nef antagonism of A17 is also dependent on its prodomain, we examined five mutants (M5, M7, M8, M11, M12) and found that Nef effectively reduced their expression (Fig. 5A), indicating that the prodomain mediates this sensitivity.

To determine the mechanism of this downregulation, we tested inhibitors of proteasomal degradation (kifunensine, eeyarestatin I, MG132, lactacystin) and lysosomal/autophagy pathways (Bafilomycin A1, 3-Methyladenine, NH_4_Cl, Concanamycin A). None of these treatments prevented Nef-induced A17 downregulation (**Fig. S8**), suggesting that Nef does not target A17 for degradation.

Using BiFC, we visualized the A17–Nef interaction in live HeLa cells. Expression of A17-VN and Nef-VC produced BiFC signals that overlapped with the fluorescence from A17, confirming the formation of an A17–Nef complex (Fig. 5B). These complexes accumulated in vacuole-like structures near the plasma membrane.

To identify their subcellular localization, we expressed mCherry-tagged cellular markers (Calreticulin, TGN46, Rab7, LAMP1, Rab11, CD63) with GFP-tagged A17 and HA-tagged Nef. Confocal microscopy showed A17 and Nef colocalized with Rab11 and CD63 in vesicle-like structures (Figs. 5C, 5D), markers for recycling endosomes and exosomes. These data suggest Nef recruits A17 into extracellular vesicles (EVs) via these pathways.

To directly detect A17 secretion, we fused a high-affinity binary NanoLuc fragment (HiBiT) to the A17 C-terminus. When expressed with Nef, A17-HiBiT was secreted into EV-enriched fractions (7 and 8) purified by size exclusion chromatography (SEC) and detected using complementation with the large Binary technology subunit (LgBiT) (Fig. 5E). In the absence of Nef, A17 secretion was significantly lower, indicating that Nef promotes A17 export via exosomes to reduce its cellular presence and neutralize its antiviral activity.

To understand the role of Nef domains in this process, we tested two Nef mutants: G2A (lacking the N-terminal myristoylation signal, required for membrane association) and ΔPxxP (lacking the SH3-binding domain). In MOLT-3 cells, the G2A mutant only replicated in *Ser5*/ *A17* double-KO cells, while the ΔPxxP mutant replicated in *A17*-KO and double-KO cells (Fig. 5F). In addition, the ΔPxxP mutant lost the ability to decrease the endogenous A17 protein expression (Fig. 5G). These results demonstrate that Nef’s myristoylation is required to antagonize both Ser5 and A17, but the SH3-binding motif is specifically required to counteract A17.

## Discussion

We have identified ADAM17 as a host restriction factor that inhibits HIV-1 infection by blocking Env expression. Our results also show that SERINC5 suppresses Env expression, but only in the context of endogenous protein. Ectopic SERINC5 expression broadly inhibits HIV-1 protein production, suggesting a context-dependent mechanism. While ADAM17 and SERINC5 physically interact, they restrict HIV-1 independently.

Though the mechanism by which SERINC5 limits Env remains unclear, we demonstrate that ADAM17 alters Env trafficking by retaining gp160 in the ER. This restriction does not require ADAM17’s metalloprotease activity but instead depends entirely on its prodomain. The antiviral function persists even without furin-mediated cleavage, indicating that the proenzyme form of ADAM17 is responsible for interacting with Env in the ER. This model is supported by co-immunoprecipitation, confocal microscopy colocalization, and AlphaFold structural predictions of the ADAM17–Env complex. Unlike SERINC5, ADAM17 inhibits a broad range of HIV-1 strains, including laboratory-adapted viruses and primary isolates of multiple subtypes, with both CXCR4 and CCR5 tropisms. This highlights ADAM17’s likely importance as a natural barrier to HIV-1 infection.

We also observed weak antiviral activity from ADAM9 and ADAM15, but not from ADAM10, contradicting a recent report that ADAM10 restricts HIV-1 by aberrantly cleaving the gp41 ectodomain in a Nef-sensitive, metalloprotease-dependent manner^15^. In our study, single knockout of *ADAM10* or *ADAM17* did not allow robust ΔNef replication in MOLT-3 cells. One key difference is that we used clonal knockout, whereas the other study used a pooled population, which may have contributed to the discrepancy. Furthermore, we were unable to restore ΔNef virus replication unless both *ADAM17* and *SERINC5* were knocked out, whereas they reported that *ADAM10* knockout alone was sufficient—results that are inconsistent with earlier findings^1,2^. We tracked ΔNef virus replication in *ADAM10*-KO cells for 11 days and observed no replication. The other group reported ΔNef virus outgrowth only after 13 days, possibly due to the strong selective pressure for restoring Nef function in these cells.

Mechanistically, Nef antagonizes SERINC5 via endolysosomal degradation, but it counters ADAM17 through a distinct process. Nef redirects ADAM17 to Rab11^+^ recycling endosomes and CD63^+^ exosomes, leading to extracellular secretion rather than degradation. This requires both the myristoylation motif and the SH3-binding domain of Nef, the latter likely activating Src family kinases such as HcK. Importantly, ADAM17’s prodomain is also essential for this pathway, consistent with our broader findings.

Our results align with earlier work showing that Nef traffics ADAM17 into extracellular vesicles^16^, but extend those findings by clarifying the molecular requirements and the functional consequence for virion infectivity. Because ADAM17 activates TNF-α during secretion into extracellular vesicles, its removal by Nef from infected cells not only lifts a potent viral restriction but may also enhance inflammatory signaling in bystander cells. Thus, Nef appears to simultaneously promote viral replication and inflammation, “killing two birds with one stone.”

## Materials And Methods

### Antibodies and inhibitors.

Inhibitor chemicals include Kifunensine (MCE, HY-19332), Eeyarestatin I (MCE, HY-110078), MG132(MCE, HY-13259), Lactacystin (MCE, HY-16594), Bafilomycin A1 (MCE, HY-100558), 3-Methyladenine (MCE, HY-19312), Concanamycin A (MCE, HY-N1724), Marimastat (MCE, HY-12169), and ammonium chloride.

Antibodies for Western blotting include mouse monoclonal anti-FLAG (1:5000 dilution) and anti-HA (1:5000 dilution) (Sigma-Aldrich, F3165, H3663), HRP-conjugated mouse monoclonal anti-GAPDH (1:5000 dilution) (Proteintech, 60004-1-Ig), HRP-conjugated goat anti-rabbit IgG antibody (1:10000 dilution), goat anti-mouse IgG antibody (1:10000 dilution), and rabbit anti-goat IgG antibody (1:10000 dilution) (Sigma-Aldrich, 12-348, 12-349, AP106), mouse monoclonal anti-ADAM17 antibody (1:1000 dilution) (Proteintech, 68725-1-Ig), rabbit polyclonal anti-ADAM17 antibody (1:1000 dilution) (Proteintech, 29948-1-AP), rabbit polyclonal anti-ADAM15 antibody (1:500 dilution) (Proteintech, 27124-1-AP), goat polyclonal anti-ADAM9 antibody (1:1000 dilution) (Bio-Techne R&D, AF949), and rabbit polyclonal anti-ADAM10 antibody (1:1000 dilution) (Abcam, ab1997).

Antibodies for flow cytometry include anti-human ADAM17 PE-conjugated antibody (1:100 dilution) (Bio-Techne R&D, FAB9301P); anti-human ADAM10 PE-conjugated antibody (1:100 dilution) (Biolegend, 352704).

Antibodies for immunofluorescence were rabbit polyclonal anti-HA (1:200 dilution) (Proteintech, 51064-2-AP) and goat anti-rabbit secondary antibody conjugated with Alexa Fluor 647 (1:500 dilution) (ThermoFisher, A-21245).

Antibodies obtained from BEI include mouse monoclonal anti-HIV-1 gp120 (1:1000 dilution) (ARP-522), mouse monoclonal anti-HIV-1 gp120 (1:1000 dilution) (ARP-2343), recombinant monoclonal anti-HIV-1 gp41 (1:1000 dilution) (ARP-1245), rabbit polyclonal anti-HIV-1 Nef (1:1000 dilution) (ARP-2949), and mouse monoclonal anti-HIV-1 p24^Gag^ (1:3000 dilution) (ARP-6458).

### Cell lines.

HEK293T cells (Cat No CRL-3216), HeLa cells (CRM-CCL-2), and A549 cells (CCL-185) were purchased from American Type Culture Collection. MOLT-3 cells (RT-12187), TZM-bI cells (HRP-8129), A549-ACE2-TMPRSS2 cells (NR-55293), GHOST (3)-CXCR4-CCR5 cells (ARP-3942) were obtained from BEI. CEM Rev-A3R5-GFP reporter cells were provided by Yuntao Wu.

HEK293T, HeLa, TZM-bI, A549, and GHOST cells were maintained in Dulbecco’s modified Eagle medium supplemented with 10% bovine calf serum (BCS) and 1% penicillin-streptomycin (pen-strep). MOLT-3, CEM, and hPBMCs were maintained in RPMI 1640 medium with 10% fetal bovine serum (FBS) and 1% pen-strep.

All these cells were and cultivated at 37°C in the humidified atmosphere in 5% CO_2_ incubators.

### Plasmids.

HIV-1 proviral vectors pNL4-3, pNL4-3ΔNef, and pMD (pNL4-3NefG2A) were reported previously^17^; pH-SF2Nef (a simplified pNL4-3 vector expressing Nef from HIV-1 SF2 strain) was reported previously^18^; pH-AD8 (a simplified pNL4-3 vector expressing Env from HIV-1 AD8 strain), pH-AD8ΔNef, pH-NL-Env-VN-HA, and pH-NL-Env-FLAG-VC were reported previously^9^; pNL4-3ΔGag and pNL4-3ΔEnv were reported previously^19^; Env-IMC.LucR infectious vectors expressing Env from HIV-1 BaL, 398F1, 246F3, CNE8, CNE55, TRO11, X2278, BJOX2000, CH1119, CE1176, 25710, CE0217, and X1632 strains were provided by Christina Ochsenbauer; pNL4-3Nef97ZA012 was provided by Henrich Gottlinger; pNLnΔBS was provided by Kenzo Tokunaga. pNL4-3NefΔPxxP was created by site-directed mutagenesis; pNL4-3ΔEnv-NLuc was constructed by replacing the *nef* gene in the pNL4-3ΔEnv vector with the NanoLuc luciferase gene via NotI/XhoI digestion.

EBOV-GP, H5N1-HA, SARS2-S, and VSV-G expression vectors were reported previously^20^; pCMV6-Ser5-FLAG and pcDNA3.1-Ser5-FLAG-VC were reported previously^21^. pcDNA3-ADAM10-HA was a gift from Axel Ullrich (Addgene plasmid # 65106; http://n2t.net/addgene:65106; RRID:Addgene_65106); pcDNA3-ADAM17-HA was a gift from Axel Ullrich (Addgene plasmid # 65105; http://n2t.net/addgene:65105;RRID:Addgene_65105). ADAM10 and ADAM17 were subcloned to generate pCMV-ADAM10-FLAG, pCMV-ADAM17-FLAG, pCMV-ADAM10-VN-HA, and pCMV-ADAM17-VN-HA by homologous recombination using DNA Assembly Master Mix (NEB, E2621). ADAM9 and ADAM15 genes were commercially synthesized to generate pCMV-ADAM9-FLAG, pCMV-ADAM15-FLAG, pCMV-ADAM15-VN-HA. ADAM17 mutants (M1-M13) were generated from pCMV-ADAM17-FLAG by Q5 site-directed mutagenesis kit (NEB, E0554) and homologous recombination. pCMV-Nef-HA was subcloned from pNL4-3 by homologous recombination; pCMV-Nef-HA-ΔPxxP was created by site-directed mutagenesis; pCMV-GFP-HA was created by PCR and homologous recombination; pcDNA3.1-Nef-FLAG-VC was constructed by replacing the *Ser5* in pcDNA3.1-Ser5-FLAG-VC with Nef (NL).

The 3^rd^-generation lentiviral vector for GFP fusion, pLJM1-GFP, was a gift from David Sabatini (Addgene plasmid # 19319; http://n2t.net/addgene:19319;RRID:Addgene_19319). Its puromycin-resistant gene was replaced by the Zeocin-resistant gene through homologous recombination. Subsequently, the GFP gene was replaced with either full-length human *ADAM17* tagged with HA (A17-HA), or its prodomain sequence tagged with HA (A17-Pro-HA), via homologous recombination, generating pLJM1-Zeocin-A17-HA and pLJM1-Zeocin-A17-Pro-HA.

mCherry-Calreticulin-N-16 was a gift from Michael Davidson (Addgene plasmid # 55006; http://n2t.net/addgene:55006;RRID:Addgene_55006); mCherry-TGNP-N-10 was a gift from Michael Davidson (Addgene plasmid # 55145; http://n2t.net/addgene:55145;RRID:Addgene_55145); mCh-Rab7A was a gift from Gia Voeltz (Addgene plasmid # 61804; http://n2t.net/addgene:61804;RRID:Addgene_61804); pLAMP1-mCherry was a gift from Amy Palmer (Addgene plasmid # 45147; http://n2t.net/addgene:45147;RRID:Addgene_45147); mCherry-Rab11a-7 was a gift from Michael Davidson (Addgene plasmid # 55124; http://n2t.net/addgene:55124;RRID:Addgene_55124); CD63-pEGFP C2 was a gift from Paul Luzio (Addgene plasmid # 62964; http://n2t.net/addgene:62964;RRID:Addgene_62964). pCMV-mCherry-CD63 was constructed by homologous recombination.

LentiCRISPRv2-puro was a gift from Feng Zhang (Addgene plasmid # 52961; http://n2t.net/addgene:52961;RRID:Addgene_52961); lentiCRISPRv2-neo was a gift from Brett Stringer (Addgene plasmid # 98292 ; http://n2t.net/addgene:98292 ; RRID:Addgene_98292); lentiCRISPRv2-blast was a gift from Brett Stringer (Addgene plasmid # 98293; http://n2t.net/addgene:98293;RRID:Addgene_98293); pCMV-VSV-G was a gift from Bob Weinberg (Addgene plasmid # 8454; http://n2t.net/addgene:8454;RRID:Addgene_8454); p8.91 was a gift from Simon Davis (Addgene plasmid # 187441; http://n2t.net/addgene:187441;RRID:Addgene_187441),

Plasmids were prepared using Plasmid Midiprep kits (Promega, A2496). All constructs were verified by Sanger sequencing.

### Transfection.

HEK293T cells were cultured in 6-well plates or 10-cm dishes and transfected with polyethyleneimine (PEI, Polysciences, 23966-2). HeLa cells were transfected using Lipofectamine 3000 according to the manufacturer’s protocol (ThermoFisher, L3000015). The total indicated plasmids were diluted into serum-free Opti-MEM (ThermoFisher, 31985062) and mixed with transfection reagents. After 15 min of incubation at room temperature, these transfection complexes were added directly into the supernatant of each well. Media were replaced after 6 hours, and cell lysate was collected at 48 hours.

### Single-cycle HIV-1 virion infectivity assay.

To measure HIV-1 infectivity, viruses were produced from HEK293T cells in 6-well plates after transfection with proviral vectors and protein expression vectors. HIV-1 virions were from the supernatants and quantified by p24^Gag^ ELISA. TZM-bI cells were seeded at 10,000 cells per well in 100 μL medium in 96-well half area transparent bottom plates (Corning, 3885). Each well was inoculated with 100 μL of viruses that were subjected to 10-fold serial dilution and infection was done in triplicate. After 48 hours, viral infection was determined by a Firefly Luciferase Assay Kit (Biotium, 30085). Virion infectivity is presented as relative light units (RLU) normalized by the p24^Gag^ levels of the inoculated viruses. Alternatively, a total of 4×10^5^ CEM Rev-A3R5-GFP cells in 2 mL medium were cultured in 6-well plates and infected with HIV-1 virions (20 ng p24^Gag^). After 48 hours, GFP^+^ cells were detected by flow cytometry.

To measure HIV-1 pseudovirion (PV) infectivity, HEK293T cells were cultured in 6-well plates and transfected with a HIV-1 proviral vector (pNL-ΔEnv-NLuc) and viral glycoprotein expression vectors including EBOV-GP, HIV-1 Env, Influenza A virus H5N1-HA and NA, SARS2-S, or VSV-G along with SERINC5, ADAM9, ADAM10, ADAM15 or ADAM17 expression vectors. Supernatants were harvested 48 hours post-transfection and centrifuged at 5000 × g for 10 min to clear cell debris and quantified by p24^Gag^ ELISA. Target cells were seeded and infected similarly to before. After 48 hours, virion infection was determined by a Renilla Luciferase Assay Kit (Biotium, 30082). Viral infectivity was calculated by normalizing luciferase activities (RLU) to the amounts of p24^Gag^ in the viral inoculum. For EBOV-GP, H5N1-HA, and VSV-G PVs, A549 cells were used as target cells; for HIV-1 Env PVs, GHOST cells were used as target cells; for SARS2-S PVs, A549-ACE2-TMPRSS2 cells were used as target cells for infection.

### CRIRPR Knockout.

DNA oligos encoding small guide RNAs (sgRNAs) targeting *SERINC5*, *ADAM9*, *ADAM10*, *ADAM15* and *ADAM17* were synthesized by Integrated DNA Technology (IDT) and cloned into LentiCRISPR v2 vectors via BsmBI digestion and homologous recombination. The targeted DNA sequences are: 5’-ATACATGATGAGCGTCACCA-3’ (SERINC5), 5’-TGTGCCCCGAATGAGGACCA-3’ (ADAM17); 5’-GATTGTGGCTGATCACTCGG-3’ (ADAM15); 5’- CGGACACGAAGGGTCCCCGA-3’ (ADAM9); 5’- GATACCTCTCATATTTACAC-3’ (ADAM10-1); 5’- GGAAATGGAATGGTAGAACA-3’ (ADAM10-2).

These lentiCRISPR vectors were co-transfected into HEK293T cells with the packaging plasmids pCMV-VSV-G and p8.91 at a 4:1:2 ratio, and after 72 hours, lentiviruses were harvested and used to infect HEK293T and MOLT-3 cells. Positive HEK293T *Ser5*-KO, HEK293T *A17*-KO, MOLT-3 *Ser5*-KO, and MOLT-3 *A17*-KO cells were isolated by selection with puromycin (2 mg/mL). HEK293T *A9*-KO, HEK293T *A1*0-KO, and HEK293T *A15*-KO cells were isolated by selection with blasticidin (5 mg/mL). HEK293T *Ser5*/ *A9* double-KO, HEK293T *Ser5*/ *A10* double-KO, HEK293T *Ser5*/ *A15* double-KO, and HEK293T *Ser5*/ *A1*7 double-KO cells were generated from HEK293T *Ser5*-KO cells and selected using blasticidin (5 mg/mL). MOLT-3 *Ser5*/ *A17* double-KO cells were generated from MOLT-3 *A17*-KO cells and selected using neomycin (1 mg/mL). MOLT-3 *Ser5*/ *A10* double-KO cells were generated from MOLT-3 *Ser5*-KO cells and selected using blasticidin (5 mg/mL). All these KO cells were subjected to single cell sorting by flow cytometry and subsequently cultured for clonal expansion. Knockout clones were identified by Next Generation Sequencing (NGS) or Western blotting (WB).

Source leukocytes were purchased from Gulf Coast Regional Blood Center. Human peripheral blood mononuclear cells (hPBMCs) were isolated and CD19^+^ cells were depleted using a CD19 Positive Selection Kit (STEMCELL, 17854). hPMBCs were cultured with 20 U/mL interleukin-2 (STEMCELL, 78145) and activated with 2.5 μg/mL phytohemagglutinin-L (PHA-L) (ThermoFisher, 00-4977). After 3 days’ activation, cells were electroporated with recombinant S. pyogenes Cas9 nuclease with GFP-tag (IDT, 10008161) and synthesized *SERINC*5/ *ADAM17* sgRNAs (IDT) by Lonza 4D-Nucleofector using P3 Primary Cell 4D X Kit (Lonza, V4XP-3012). After 18 hours, GFP positive cells were sorted by flow cytometry for HIV-1 infection.

### Reconstitution of MOLT-3 cell lines.

Lentiviruses were produced from HEK293T cells after transfection with pLJM1-Zeocin-A17-HA or pLJM1-Zeocin-A17-Pro-HA and the packaging vectors pCMV-VSV-G and p8.91 at a 4:1:2 ratio. MOLT-3 *Ser5*/ *A17* double-KO cells were infected and selected by Zeocin (100 mg/mL).

### Next Generation Sequencing (NGS).

The genomic DNA from the target cells were extracted using the Genomic DNA Kits (Promega, PAS1880). Then the sgRNA targeted genome region was ampliified using NGS sequencing primers with a unique barcode, Forward Primer: 5’-ACACTGACGACATGGTTCTACACTCTCCCTCTTACCCACTTCCCG-3’ and Reverse Primer: 5’-TACGGTAGCAGAGACTTGGTCTCCCACCTTGCCTACTGCTGAC-3’. The amplicons were sequenced on Illumina MiSeq (V3).

### HIV-1 Infection of MOLT-3 and hPBMCs.

A total of 2×10^5^ MOLT-3 cells and 1×10^6^ activated hPBMCs were cultured in 5 mL medium and infected with HIV-1 (1 or 4 ng p24^Gag^). Cells were cultured for 9-11 days, and culture supernatants were then collected every other day for measurement by p24^Gag^ ELISA.

### Immunoprecipitation.

After transfection of HEK293T cells cultured in a 10-cm dish, cells were lysed in 800 μL IP lysis buffer (ThermoFisher, 87787) containing 1% protease inhibitors (ThermoFisher, 87785) for 30 min on ice. After the removal of nuclei via low-speed centrifugation and collecting 100 μL as input, the remaining 700 μL lysate was incubated with anti-FLAG M2 Magnetic beads (Sigma-Aldrich, M8823) or anti-HA Magnetic Beads (Sigma-Aldrich, SAE0197) and rotated at 4 °C overnight. After washing 3 times with 1 mL pre-cooled IP lysis buffer, proteins were removed from beads using competitive 3× FLAG Peptide (Sigma-Aldrich, F4799) or boiled in sample loading buffer.

### Confocal microscopy.

HeLa cells were seeded on a glass bottom cell culture dish (ThermoFisher, 177402PK) and transfected with various vectors using Lipofectamine 3000. After 30 hours, cells were fixed with 4% paraformaldehyde for 10 min, permeabilized with 0.1% Saponin (Sigma-Aldrich, SAE0073) for 10 min at room temperature, and then blocked with Immunofluorescence Blocking Buffer (CST, 12411) at 37 °C for 30 min. The samples were stained with primary antibody at 4 °C overnight, washed with PBS 3 times. Cells were then stained with the secondary antibody at 37°C for 1 hour and washed with PBS 3 times. Nuclei were stained with 4′,6-diamidino-2-phenylindole (DAPI) for 3-5 min. After being washed 3 times with PBS, cells were observed and imaged under a confocal microscope (Zeiss, LSM 980 AIRYSCAN 2). Quantitative colocalization measurements were performed using ImageJ software.

### Western blotting (WB).

Typically, HEK293T cells were seeded in 6-well plates or in 10-cm dishes with an initial density of 1 × 10^6^ cells per well or 3 × 10^6^ cells per dish. Transfected cells were lysed in RIPA buffer (ThermoFisher, 89900) containing 1% protease Inhibitor (ThermoFisher, 87785) on ice for 30 min. After centrifugation at 12,000 × g for 10 min at 4°C, cytosolic fractions were collected and mixed with 4 × Laemmli Sample Buffer (Biorad, 1610747) containing 50 mM dithiothreitol (BioRad, 1610610). Proteins were separated by SDS-PAGE and transferred onto PVDF membranes. These membranes were blocked with 5% nonfat milk powder in TBST (Tris-buffered saline [20 mM Tris, pH 7.4,150 mM NaCl] containing 0.1% Tween 20) for 1 hour at room temperature and probed by primary antibodies followed by HRP-conjugated secondary antibodies. Chemiluminescence signals were produced by incubating the membrane with Immobilon Classico Western HRP substrate (Millipore, WBLUC0500), and detected by an Imaging System (ThermoFisher, iBright 1500).

### Flow cytometry.

HEK293T and MOLT-3 knockout cells were washed twice with PBS and sorted into 96-well plates as single clones using Beckman MOFLO ASTRIOS. hPBMCs nucleofected with Cas9-GFP RNPs were washed twice with PBS, and GFP^+^ cells were sorted out similarly using the 488 nm excitation channel. HIV-1 infected Rev-A3R5-GFP reporter cells were washed with PBS and fixed with 4% paraformaldehyde for 30 min. GFP^+^ cells were analyzed by Beckman MOFLO ASTRIOS. To detect ADAM10 and ADAM17 expression, MOLT-3 cells or hPBMCs were washed twice with PBS containing 2% FBS. Cells were incubated with PE-conjugated anti-human ADAM17 or ADAM10 antibodies for 30 min at 4°C in the dark. After being washed, cells were resuspended in PBS with 2% FBS and analyzed similarly. Positive cells were gated based on fluorescence intensity relative to an isotype control, and data were analyzed using FlowJo.

### Mass Spectrometry.

HEK293T cells were transfected with the Ser5-FLAG and/or Nef-HA expression vectors. After 24 hours of transfection, cells were lysed with RIPA buffer. The cell lysate was incubated with anti-FLAG M2 magnetic beads or anti-HA Affinity Gel for 24 hours at 4 °C. The beads and the gel were then washed with PBS five times and mixed with protein loading buffer and electrophoresed in SDS-PAGE. The gel was then stained with Coomassie blue and sent to the Laboratory of Proteomics, Institute of Biophysics, Chinese Academy of Sciences, for Nano LC-MS/MS and database search analysis. The raw data can be downloaded from iProX (project ID: IPX0010260000).

### Isolation of extracellular vesicles (EVs).

HEK293T cells were transfected with pCMV-ADAM17-HiBiT and pCMV-Nef-HA. After 48 hours, supernatants were collected and centrifuged at 4,000 × g for 20 min at 4°C to remove cellular debris. Clearified supernatants were concentrated using 30kDa Amicon 15 mL filters (Sigma, UFC903008) by centrifugation at 2,500 × g for 60 min at 4°C. The concentrated supernatant was subjected to size exclusion chromatography (SEC) using 35 nm SEC column (IZON, ICO-35). Fractions were lysed and A17-HiBiT was quantified using the HiBiT Detection Kit (Promega, N3040).

### Prediction of Protein Complexes and Identification of Binding Interfaces.

We used AlphaFold3^22^ to predict the protein complexes of ADAM17 (UNIPROT: P78536) and HIV-1 gp160 (Q75760). To define the protein-protein binding interface in the predicted complexes, we employed the alpha shape analysis^23^. A detailed interface is shown in Table S1.

## Supplementary Material

This is a list of supplementary files associated with this preprint. Click to download.

SupplementalInformation.pdf

## Data Availability

Source data are provided with this paper.
